# Exploring the Association Between Cognitive Decline and Triglyceride‐Glucose Index: A Systematic Review and Meta‐Analysis

**DOI:** 10.1002/brb3.70131

**Published:** 2024-10-31

**Authors:** Elina Ghondaghsaz, Amirmohammad Khalaji, Mehrdad Mahalleh, Mahdi Masrour, Parsa Mohammadi, Alessandro Cannavo, Amir Hossein Behnoush

**Affiliations:** ^1^ Undergraduate Program in Neuroscience University of British Columbia British Columbia Vancouver Canada; ^2^ School of Medicine Tehran University of Medical Sciences Tehran Iran; ^3^ Non‐Communicable Diseases Research Center Endocrinology and Metabolism Population Sciences Institute Tehran University of Medical Sciences Tehran Iran; ^4^ Rheumatology Research Center Tehran University of Medical Sciences Tehran Iran; ^5^ Department of Translational Medicine Sciences Federico II University of Naples Naples Italy

**Keywords:** cognitive decline, cognitive impairment, dementia, meta‐analysis, systematic review, triglyceride‐glucose index

## Abstract

**Background:**

Cognitive decline and dementia are debilitating conditions that compromise the quality of life and charge the healthcare system with a substantial socioeconomic burden. In this context, emerging evidence supports an association between the triglyceride‐glucose index (TyG), a surrogate insulin resistance marker, and cognitive decline and dementia. Hence, we systematically reviewed the studies assessing the TyG index in patients with cognitive decline and their controls.

**Methods:**

Online international databases (PubMed, Scopus, Embase, and the Web of Science) were searched comprehensively for studies showing the TyG index in patients with cognitive decline/impairment. Random‐effect meta‐analyses were conducted to calculate the standardized mean difference (SMD), pooled odds ratio (OR), and pooled area under the curve (AUC), in addition to 95% confidence intervals (CIs) for the comparisons of groups.

**Results:**

Seventeen studies were included in our analysis. Then, we conducted a meta‐analysis, demonstrating that patients with cognitive decline had significantly higher levels of TyG index than those without (SMD 0.83, 95% CI 0.16 to 1.50, *p* = 0.015). Moreover, our data showed that a 1‐unit increase in the TyG index was associated with higher odds of cognitive decline (adjusted OR [aOR] 2.86, 95% CI 1.49 to 5.50, *p* = 0.002). Further, we observed that patients in the fourth TyG quartile with higher values of the TyG index than the first quartile presented with more increased cognitive decline (aOR 1.62, 95%CI 1.11 to 2.38, *p* = 0.013). Finally, pooled AUC data for the diagnostic performance of the TyG index resulted in an overall AUC value of 0.73 (95% CI 0.66 to 0.79). Sensitivity and specificity were also calculated as 0.695 and 0.687, respectively.

**Conclusion:**

This study supports the clinical utility of the TyG index in patients with cognitive decline and solicits more focused studies to consolidate its usage in clinical settings and real‐world practice.

## Graphical abstract

The role of insulin resistance and triglyceride‐glucose index (TyG) has been investigated in cognitive decline and dementia. Based on 17 included studies, patients with cognitive decline had significantly higher levels of TyG index compared to controls. TyG, as a surrogate marker of insulin resistance, could be a potentially useful biomarker in dementia and cognitive decline.

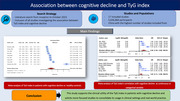

## 1 Introduction

Dementia is an “umbrella” term that generally describes several brain diseases affecting memory, other cognitive abilities, and behavior that significantly decrease individuals’ quality of life, along with their families, with substantial social and economic burdens (WHO 2023). There are four common types of dementia: (1) Alzheimer's disease, accounting for 60%–80% of all cases; (2) vascular dementia, which is the second most prevalent form with 20% of patients affected; (3) Lewy body dementia, with 5%–15% of all dementias, represents the third form of dementia and includes two subtypes of Parkinson's disease and Lewy body dementia; (4) the less common form defined by frontotemporal dementia.

Overall, dementia represents a significant and growing global health challenge, with almost 50 million people currently affected, according to the World Health Organization (WHO 2023; Wu et al. 2017; GBD 2016 Dementia Collaborators 2019). Although it is not a normal stage of the aging process, age is considered the most potent known risk factor for dementia. Therefore, it has been estimated that due to the increasing geriatric population, the global total of people with dementia will double in 2030 and triplicate in 2050 (WHO 2023; Kong et al. 2018). Based on this premise, accurate diagnosis and the identification of modifiable risk factors are two prerequisites for delivering optimal therapies specific to diverse dementia subtypes that can help to reduce its global burden. In this context, understanding cognitive decline is one of the most critical issues (Jongsiriyanyong and Limpawattana 2018). The spectrum of cognitive decline ranges from an average cognitive decline observed with aging to mild cognitive impairment (MCI), an intermediate state, to dementia (Jongsiriyanyong and Limpawattana 2018). The term MCI is used when the decline in cognition is higher than expected for one's age and education level without fulfilling the dementia criteria (Petersen et al. 2018). The likelihood of progression from MCI to any of the types of dementia has been estimated to range from 5% to 17% per year, and the risk factors include age, MCI subtype, brain imaging characteristics like reduced volume or increased white matter intensities (WMI), cerebrospinal fluid (CSF) biomarkers, and lack of social engagement (Petersen et al. 2018). In addition, the presence of concurrent conditions such as heart failure and depression in which insulin resistance (IR) plays a role might have associations with cognitive decline (Tudoran et al. 2020). Therefore, much focus has been on identifying modifiable and nonmodifiable risk factors to prevent the transition from MCI to dementia (Jongsiriyanyong and Limpawattana 2018).

The Lancet Commission on Dementia Prevention, Intervention, and Care has reported diabetes among a list of independent risk factors for dementia (Livingston et al. 2020). Despite this, how diabetes, cognitive decline, and dementia are entangled and whether these disorders share a common pathogenic mechanism remain two big unresolved questions. Importantly, several pieces of evidence have identified IR as a probable mechanistic link factor in this association. In addition, IR acts as a metabolic substrate of several other independent risk factors of dementia (Livingston et al. 2020), supporting the idea that this condition plays a direct pivotal role in cognitive decline and dementia. This idea has been corroborated also by observation of a relationship between IR and cognitive impairment in both diabetic and nondiabetic populations (Benedict et al. 2012; Backeström et al. 2015; Lutski et al. 2017; Wei et al. 2023; Kim and Arvanitakis 2023). Despite this evidence, several gaps remain. Therefore, by our previous report and in line with others, we focused on the studies assessing the triglyceride‐glucose (TyG) index, a surrogate marker of IR. The TyG index is easily calculated using fasting triglycerides and fasting plasma glucose values and compared with other tools to assess IR (Sánchez‐Íñigo et al. 2016; Khalaji et al. 2023; Okamura et al. 2020; Hong, Han, and Park 2021). This index presents high sensitivity and specificity and can be used as a helpful index of diabetes and several other IR‐related disorders, including dementia and depression (Sánchez‐Íñigo et al. 2016; Khalaji et al. 2023; Okamura et al. 2020; Hong, Han, and Park 2021; Behnoush et al. 2024). Based on this premise, we conducted a systematic review and meta‐analysis to establish if the TyG index is an accessible predictor of cognitive decline and dementia.

## 2 Methods

The systematic review and meta‐analysis were conducted in adherence to the Preferred Reporting Items for Systematic Reviews and Meta‐Analyses (PRISMA) guidelines (Page et al. 2021). The protocol of this study was officially documented in the International Prospective Register of Systematic Reviews (PROSPERO) with the registration number CRD42023475580 on November 8, 2023.

### 2.1 Literature Search

A systematic search was carried out on four online databases, including PubMed, Web of Science, Scopus, and Embase, on October 25, 2023, to identify the most relevant publications. No limitations were placed on the year of publication or any other filters. A combination of Medical Subject Headings (MeSH) terms and free‐text keywords related to “TyG” and “cognitive impairment,” along with their corresponding expansions, were utilized to query the databases. The search query is provided in Table S1.

### 2.2 Selection Criteria

Our study incorporated original research that fulfilled one of the following criteria: (1) it presented data on the TyG index in individuals with cognitive decline and in controls; (2) it assessed the diagnostic precision of the TyG index in distinguishing between individuals with cognitive decline and controls, using measures such as sensitivity, specificity, and area under the curve (AUC); (3) it reported the correlation between the TyG Index levels and the prevalence of cognitive decline, either in the form of odds ratios (ORs) or hazard ratios (HRs); (4) it examined the predictive capability of categorized TyG index values in identifying concurrent cognitive decline; and (5) population‐based studies on healthy subjects that assessed the effect of TyG on the risk of cognitive decline or dementia development. Exclusion criteria encompassed review articles, case reports, and non‐English publications lacking English abstracts. There was no restriction on date of publication among studies, and all studies from inception to search date were included.

### 2.3 Data Extraction

The information from the included studies was collected independently by two investigators, AK and EG, using an electronic spreadsheet. The relevant information extracted from each study, when available, contained the authors' names, year of publication, country of origin, study design, sample size, control population, mean age, male percentage, TyG levels in different groups, diagnostic performance measures such as sensitivity, specificity, and AUC, as well as the correlation of TyG levels with concomitant cognitive decline, presented as ORs and HRs. In addition to these findings, the corresponding 95% confidence intervals (CIs) or standard deviations (SDs) and *p*‐values were also obtained. Disagreements were effectively resolved through the process of engaging in productive discussion and reaching a consensus.

### 2.4 Quality Assessment

The quality assessment of cohort and case‐control studies included in the analysis was conducted using the Newcastle‐Ottawa Scale (NOS) (Wells et al. 2000). The quality of each study was assessed independently by two investigators, AK and EG, using predetermined criteria. Discrepancies pertaining to the evaluation of quality were resolved by means of communication or consultation with an additional reviewer. The NOS comprises three primary types of bias, namely selection bias, comparability bias, and outcome bias. Ratings of “good,” “fair,” and “poor” were assigned to the categories of seven and above, two to six, and one and below, respectively.

### 2.5 Statistical Analysis

All statistical analyses and visualizations were conducted using R version 4.2.2 (R Core Team [2021], Vienna, Austria) with the assistance of the “meta” and “mada” packages (Balduzzi, Rücker, and Schwarzer 2019; Doebler and Holling 2017). The bivariate random effect model proposed by Reitsma et al. (2005) was utilized to aggregate findings from studies addressing diagnostic specificity and sensitivity. The model also computes the summary receiver operating characteristic (sROC) curve and the corresponding AUC (Reitsma et al. 2005). For the AUC values provided by the studies themselves, a meta‐analysis was conducted using the random effects model with the inverse variance method to compute a pooled AUC (Higgins, Thompson, and Spiegelhalter 2009). In order to compare the mean TyG index levels in the control groups and the groups experiencing cognitive decline, we employed the bias‐corrected Hedges' g standardized mean difference (SMD) in meta‐analysis. Hedges' g was chosen as it accounts for both test and control sample sizes when determining the effect size (Hedges 1981). A meta‐analysis using the random effects model with the inverse variance method was also performed on ORs gathered from studies that examined the TyG index as a continuous variable. The ORs in these studies reflect the risk per one‐unit increment of the TyG index. Furthermore, if the TyG index was treated as a categorical variable with four quartiles of TyG levels, the ORs for comparing the group with the highest TyG index (fourth quartile) to the group with the lowest TyG index (first quartile) were used in the meta‐analysis. Each quartile is defined according to the TyG levels in each population. The first quartile (Q1) is the first 25% of data, and the fourth quartile (Q4) is the last 25%. Unadjusted and most adjusted OR values were analyzed and reported separately for the two mentioned meta‐analyses.

The random effects model was employed in all analyses due to the prediction of high heterogeneity between the analyzed studies and the marginally different measurement techniques they used. I2 and tau2 statistics were employed for the assessment of heterogeneity in all meta‐analyses. Multivariant meta‐regression was also used to investigate the sources of heterogeneity among the studies.

When the median and interquartile range were reported, the mean and standard deviation were calculated using methods suggested by Luo and Wan (Wan et al. 2014; Luo et al. 2018). The standard error of the AUCs for use in meta‐analysis was calculated using the 95% CI. In case no CI was provided, the method developed by Hanley and McNeil was implemented for the calculation of the standard error from the sample size and the AUC (Hanley and McNeil 1982; Obuchowski, Lieber, and Wians 2004). Furthermore, a statistically significant result was considered as having a *p*‐value less than 0.05 and an I2 value greater than 50%.

## 3 Results

### 3.1 Search and Baseline Characteristics of Included Studies

**FIGURE 1:**
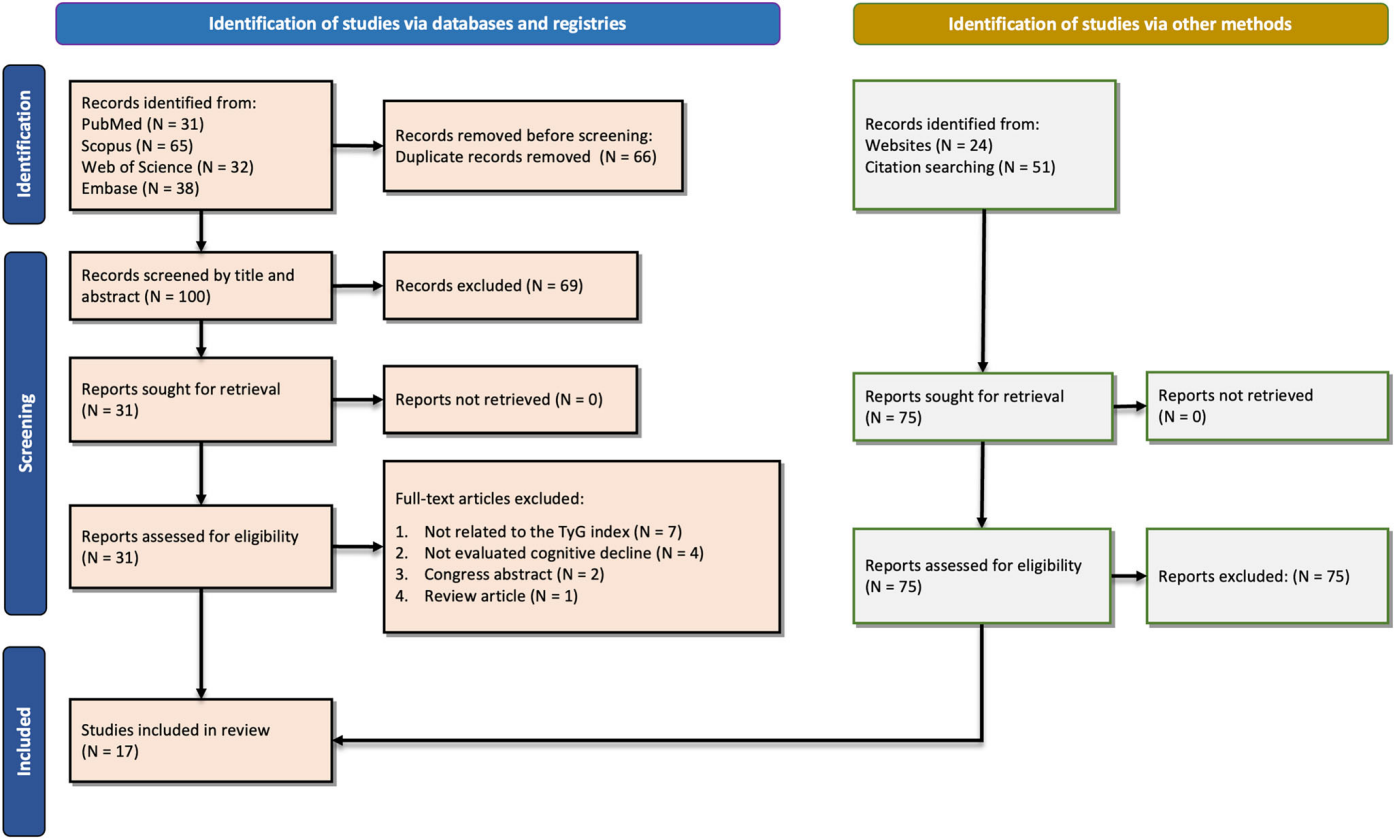
PRISMA flowchart for search, screening, and reasons for exclusion.

The screening of databases led to the identification of 166 studies, with most studies from Scopus (*n* = 65), followed by Embase (*n* = 38), the Web of Science (*n* = 32), and PubMed (*n* = 31). Subsequently, 66 studies were removed because of duplicates, whereas 83 were excluded after title/abstract and full‐text screening. As shown in Figure 1, in the PRISMA flowchart, we finally included 17 studies for the analysis (Hong, Han, and Park 2021; Faqih et al. 2021; Gentreau et al. 2022; Guo et al. 2021; Huang et al. 2022; Jiang et al. 2021; S. Li, Deng, and Zhang 2022; Liu et al. 2023; Ma et al. 2023; Seo et al. 2023; Sun et al. 2023; Teng et al. 2022; Tian, Fa et al. 2023; Tian, Song et al. 2023; Tong et al. 2022; Wang et al. 2022; Weyman‐Vela et al. 2022).

Table 1 illustrates the characteristics of the included studies on 5,636,864 participants. All the studies were published between the years 2021 and 2023 and were mainly conducted in China (Guo et al. 2021; Jiang et al. 2021; S. Li, Deng, and Zhang 2022; Liu et al. 2023; Ma et al. 2023; Sun et al. 2023; Teng et al. 2022; Tian, Fa et al. 2023; Tian, Song et al. 2023; Tong et al. 2022; Wang et al. 2022). The most extensive study was conducted in South Korea on a population of 5,568,048 individuals (Hong, Han, and Park 2021). The result of the quality assessment for studies is shown in Table S2. Based on the NOS system, all studies had high qualities.

**TABLE 1:** Baseline characteristics of the studies evaluating the TyG index in cognitive impairment.

Study	Year	Location	Population	Outcome	Sample size	Mean age (years)	Male (%)	TyG index	Main findings
Faqih et al. (2021)	2021	Saudi Arabia	Patients with AD or memory loss	AD	354	80.5 ± 10.2	46.3	NR	Higher odds of AD were reported in patients with IR compared to the non‐IR group in the age‐adjusted model (OR 1.4, 95% CI 1.01 to 2.33, *p* < 0.05).
Gentreau et al. (2022)	2022	France	Patients who developed MCI or dementia in 7‐year follow‐up and CN controls	Prodromal dementia	497	71.0 ± 3.9	48.7	8.42 ± 0.48	Comparable levels of the TyG index were found between CN individuals and the MCI/dementia group (CN: 8.41 ± 0.47, dementia/MCI: 8.45 ± 0.50, *p* = 0.706).
Guo et al. (2021)	2021	China	Elderly patients with CSVD with or without VCI	VCI	275	NR	NR	8.95 ± 0.54	The TyG index was significantly higher in the VCI group compared to the non‐VCI group (9.07 ± 0.54 vs. 8.70 ± 0.55, *p* < 0.01).
Hong, Han, and Park (2021)	2021	Republic of Korea	Population‐based study	Dementia, AD, and VD	5,586,048	44.9 ± 13.2	50.7	NR	Individuals in Q2, Q3, and Q4 of the TyG index had higher HR for dementia, AD, and VD, compared to Q1 (*p* < 0.05).
Huang et al. (2022)	2022	Taiwan	Population‐based study	MMSE ≥ 24	28,486	63.9 ± 2.9	39.2	8.5 ± 0.6	The TyG index was significantly higher in individuals with MMSE < 24 compared to those with MMSE ≥ 24 (8.6 ± 0.6 vs. 8.5 ± 0.6, *p* < 0.001).
Jiang et al. (2021)	2021	China	CSVD patients with or without VCI	VCI	280	67.6 ± 11.8	57.8	9.16 ± 0.71	The TyG index was significantly higher in the VCI group compared to the non‐VCI group (*p* < 0.001).
Li, Deng, and Zhang (2022)	2022	China	Population‐based study	Cognitive decline (MMSE)	1774	53.5 ± 8.5	48.0	8.31 ± 0.62	A higher incidence of cognitive decline was found in higher quartiles of the TyG index (*p* = 0.017).
Liu et al. (2023)	2023	China	Elderly patients with MoCA ≥ 18	MCI (MoCA)	262	53.0 ± 7.5	NR	NR	A negative correlation between the TyG index and MoCA score was found (*r* = −0.75, 95% CI −1.29 to 0.20, *p* < 0.05).
Ma et al. (2023)	2023	China	Population‐based study	Cognitive impairment (MMSE)	1484	58.1 ± 9.2	40.0	8.69 ± 0.57	Cognitively impaired individuals had significantly higher TyG index compared to CN ones (8.89 ± 0.58 vs. 8.68 ± 0.56, *p* = 0.001).
Seo et al. (2023)	2023	United States	Male firefighters aged 20–60 years	PVT and DMS	114	39.4 ± 2.6	100	8.7 ± 0.1	Significant positive correlations between DMS total time with the TyG index (*p* < 0.01) and DMS reaction time with the TyG index were found (*p* < 0.01).
Sun et al. (2023)	2023	China	Population‐based study	AD	2170	63.0 ± 8.2	46.7	8.7 ± 0.6	Baseline TyG index was associated with significantly higher risk of AD (HR 1.28, 95% CI 1.03 to 1.60, *p* = 0.027).
Teng et al. (2022)	2022	China	Elderly patients with T2DM	Cognitive impairment (MMSE)	308	70.6 ± 6.1	48.7	8.99 ± 0.68	T2DM patients with cognitive impairment had significantly higher TyG index compared to T2DM patients with no cognitive impairment (9.20 ± 0.72 vs. 8.79 ± 0.57, *p* < 0.001).
Tian, Fa et al. (2023)	2023	China	Population‐based study	Dementia, AD, and VD	5199	71.8 ± 5.5	42.9	8.62 ± 0.53	Patients with dementia had significantly higher levels of TyG index compared to those without dementia (8.73 ± 0.57 vs. 8.61 ± 0.53, *p* = 0.001)
Tian, Song et al. (2023)	2023	China	Population‐based study	Cognitive function (z‐score)	4541	71.0 ± 4.7	43.6	8.61 ± 0.53	A significant J‐shaped inverted association between the TyG index and z‐scores of verbal fluency, global cognition, and executive function were found (*p* < 0.05).
Tong et al. (2022)	2022	China	T2DM patients with or without MCI	MCI	517	58.0 ± 8.9	54.4	9.37 ± 0.70	Patients with MCI had significantly higher TyG levels compared to CN ones (9.69 [IQR 9.35–10.10] vs. 9.05 [95% CI 8.62–9.39], *p* < 0.01). Moreover, mean TyG‐BMI index was also higher in this group, compared to CN patients (246 [IQR 222–270] vs. 228 [IQR 204–249], *p* < 0.01).
Wang et al. (2022)	2022	China	Population‐based study	WRT and MST	4420	58.9 ± 8.7	46.6	8.63 ± 0.61	In males, the higher odds of cognitive decline (global cognition) were found in Q4 of TyG index compared to Q1 (reference) (OR 1.32, 95% CI 1.03 to 1.71, *p* = 0.031).
Weyman‐Vala et al. (2022)	2022	Mexico	Participants with or without MCI aged 60–90 years	MCI (MMSE)	135	72.8 ± 6.2	18.5	4.53 ± 0.25	The higher TyG index was in patients with MCI compared to CN individuals (5.0 ± 0.3 vs. 4.1 ± 0.2, *p* < 0.001).

Abbreviations: AD: Alzheimer's disease, BMI: body mass index, CI: confidence interval, CN: cognitively normal, CSVD: cerebral small vessel disease, DMS: delayed‐match‐to‐sample task, HR: hazard ratio, IQR: interquartile range, IR: insulin resistance, MCI: mild cognitive impairment, MMSE: Mini‐Mental State Examination, MoCA: Montreal Cognitive Function Scale, MST: mental status, NR: not reported, OR: odds ratio, PVT: psychomotor vigilance task, T2DM: type 2 diabetes mellitus, TyG: triglyceride‐glucose index, VCI: vascular cognitive impairment, WRT: word recall test.

### 3.2 Meta‐Analysis of the TyG Index in Patients with Cognitive Decline vs. Controls

**FIGURE 2:**
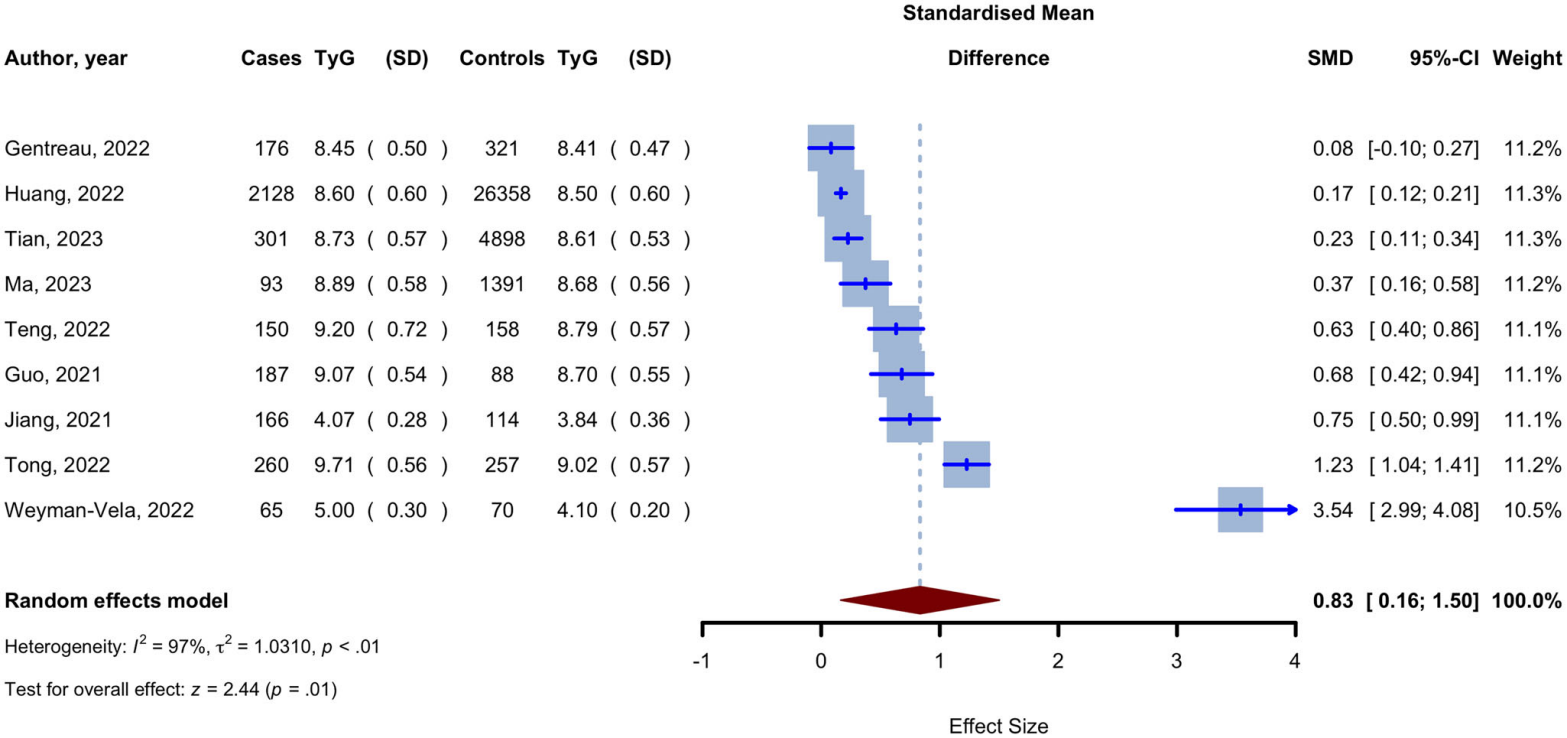
Forest plot for meta‐analysis of TyG levels in patients with cognitive decline versus controls.

Nine studies investigated the TyG index in patients with cognitive decline and compared them with controls (Gentreau et al. 2022; Guo et al. 2021; Huang et al. 2022; Jiang et al. 2021; Ma et al. 2023; Teng et al. 2022; Tian, Fa et al. 2023; Tong et al. 2022; Weyman‐Vela et al. 2022). Random‐effect meta‐analysis was performed to pool these comparisons, and as shown in the forest plot in Figure 2, patients with cognitive decline had significantly higher levels of the TyG index (SMD 0.83, 95% CI 0.16 to 1.50, *p* = 0.015). This analysis was associated with high heterogeneity (*I* ^2^ 97%, 95% CI 96% to 98%).

Sensitivity analysis by the leave‐one‐out method was performed for this meta‐analysis, and no significant change was observed by the removal of any of the studies (Figure S1). Next, we assessed publication bias using the trim‐and‐fill method and observed an asymmetry for the analysis (Figure S2). However, adding three additional studies to gain symmetry led to an insignificant difference between the cognitive decline group and controls (SMD 0.23, 95% CI −0.55 to 1.01, *p* = 0.58). Similarly, Begg's and Egger's statistical tests showed a significant publication bias (*p* = 0.022 and 0.020, respectively). Finally, multivariate meta‐regression showed that publication year, sample size, and male ratio were significantly associated with the observed pooled estimate (all *p* < 0.05). The combination of these variables and the mean age accounted for 69.48% of the heterogeneity (*R* ^2^: 69.48%, Table 2).

**TABLE 2:** Meta‐regression for meta‐analysis of TyG index in patients with cognitive decline vs. controls.

Multivariant meta‐regression				
Moderator	No. of studies	Meta‐regression slope	95% CI	*p*‐value
**Publication year**	8	−0.9871	−1.7847 to −0.1896	0.0153
**Sample size**	8	−0.0001	−0.0001 to −0.0000	0.0234
**Mean age**	8	−0.0424	−0.1268 to 0.0421	0.3255
**Male ratio**	8	−9.3006	−13.9735 to −4.6276	< 0.0001

### 3.3 Meta‐Analysis of Cognitive Decline Risk and the TyG Index

**FIGURE 3:**
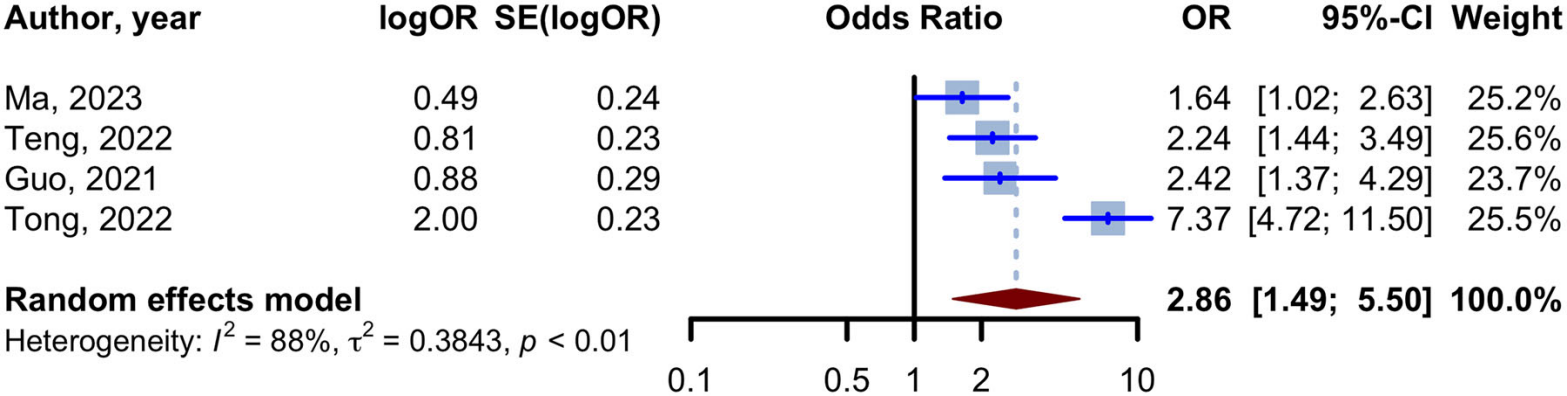
Forest plot for meta‐analysis of adjusted odds ratio for the relationship between TyG index (1‐unit increase) and cognitive decline.

The TyG index was assessed as a continuous variable in increasing the risk of cognitive decline in five studies (Guo et al. 2021; Ma et al. 2023; Sun et al. 2023; Teng et al. 2022; Tong et al. 2022). Among these, only the study by Sun et al. reported HRs for incident AD and, therefore, was not included in the meta‐analysis. Importantly, this study failed to find an association between the TyG index and the risk of AD (aHR 1.32, 95% CI 0.98 to 1.77). Next, we pooled the remaining four studies and included them in the meta‐analysis as they reported adjusted ORs for a 1‐unit increase in the TyG index (Guo et al. 2021; Ma et al. 2023; Teng et al. 2022; Tong et al. 2022). The adjustments for each of the studies are shown in Table S3. As shown in the forest plot in Figure 3, we observed that an increase in the TyG index resulted in significantly higher odds of cognitive decline (aOR 2.86, 95% CI 1.49 to 5.50, *p*‐value = 0.002) with high heterogeneity (*I* ^2^ 88%, 95% CI 70% to 95%). Random‐effect meta‐analysis (Figure S3) of unadjusted ORs showed significantly higher odds of cognitive decline with one unit increase in the TyG index (unadjusted OR 3.16, 95% CI 1.58 to 6.31, *p* = 0.001, *I* ^2^: 91%). In Table 3, we summarized ORs and HRs of cognitive decline per 1‐unit increase in the TyG index.

**TABLE 3:** Outcomes in different groups/levels of the TyG index.

Study	Population	Outcome	Group 1	Group 2	Group 3	Group 4	Continuous
Faqih et al. (2021)	Patients with AD or memory loss	AD	Low‐TyG (no IR) Ref —	High‐TyG (IR) aOR 1.2 [95% CI 1.0 to 3.1]^*^	−	—	—
Guo et al. (2021)	Elderly patients with CSVD with or without VCI	VCI	Low‐TyG (< 8.78) Ref —	High‐TyG (≥ 8.78) aOR 4.09 [95% CI 2.18 to 7.68]^*^	—	—	aOR 2.42 [95% CI 1.37 to 4.29]^*^
Hong, Han, and Park (2021)	Population‐based study	All‐cause dementia	Q1 Ref —	Q2 aHR 1.04 [95% CI 1.02 to 1.05]^*^	Q3 aHR1.07 [95% CI 1.05 to 1.09]^*^	Q4 aHR 1.14 [95% CI 1.12 to 1.16]^*^	—
Jiang et al. (2021)	CSVD patients with or without VCI	VCI	Q1 (≤ 3.77) Ref —	Q2 (3.77–4.00) aOR 2.69 [95% CI 1.17 to 6.16]^*^	Q3 (4.00–4.18) aOR 2.54 [95% CI 1.12 to 5.75]^*^	Q4 (≥ 4.18) aOR 4.67 [95% CI 1.79 to 12.16]^*^	—
Li, Deng, and Zhang (2022)	Population‐based study	Cognitive decline	Q1 (< 7.87) Ref —	Q2 (7.87–8.25) aOR 1.17 [95% CI 0.85 to 1.62]	Q3 (8.25–8.68) aOR 1.31 [95% CI 0.93 to 1.83]	Q4 (≥ 8.68) aOR 1.51 [95% CI 1.06 to 2.14]^*^	—
Ma et al. (2023)	Population‐based study	Cognitive impairment	Q1 (< 8.30) Ref —	Q2 (8.30–8.64) aOR 2.02 [95% CI 0.94 to 4.34]	Q3 (8.65–9.05) aOR 2.26 [95% CI 1.06 to 4.84]^*^	Q4 (> 9.05) aOR 2.64 [95% CI 1.19 to 5.85]^*^	aOR 1.64 [95% CI 1.02 to 2.63]^*^
Sun et al. (2023)	Population‐based study	AD	Q1 (< 8.29) Ref —	Q2 (8.29−8.67) aHR 1.59 [95% CI 0.97 to 2.62]	Q3 (8.68−9.09) aHR 1.69 [95% CI 1.02 to 2.81]^*^	Q4 (> 9.09) aHR 1.39 [95% CI 0.80 to 2.41]	aHR 1.32 [95% CI 0.98 to 1.77]
Teng et al. (2022)	Elderly patients with T2DM	Cognitive impairment	T1 (≤ 8.71) Ref —	T2 (8.72‐9.21) aOR 1.75 [95% CI 0.93 to 3.30]	T3 (≥ 9.22) aOR 3.30 [95% CI 1.68 to 6.45]^*^	—	aOR 2.24 [95% CI 1.44 to 3.49]^*^
Tian, Fa et al. (2023)	Population‐based study	All‐cause dementia	< 75^th^ percentile TyG Ref —	≥ 75^th^ percentile TyG aOR 1.66 [95% CI 1.24 to 2.20]^*^			
Tong et al. (2022)	T2DM patients with or without MCI	MCI	—	—	—	—	aOR 7.37 [95% CI 4.72 to 11.50]^*^
Wang et al. (2022)	Population‐based study (Female)	Cognitive decline	Q1 Ref —	Q2 aOR 1.13 [95% CI 0.88 to 1.45]	Q3 aOR 1.01 [95% CI 0.79 to 1.29]	Q4 aOR 1.11 [95% CI 0.87 to 1.42]	—
Wang et al. (2022)	Population‐based study (Male)	Cognitive decline	Q1 Ref —	Q2 aOR 1.09 [95% CI 0.85 to 1.40]	Q3 aOR 1.09 [95% CI 0.85 to 1.41]	Q4 aOR 1.32 [95% CI 1.03 to 1.71]^*^	—

Abbreviations: AD: Alzheimer's disease, aHR: adjusted hazard ratio, aOR: adjusted odds ratio, CI: confidence interval, CSVD: cerebral small vessel disease, IR: insulin resistance, MCI: mild cognitive impairment, Q: quartile, T: tertile, T2DM: type 2 diabetes mellitus, VCI: vascular cognitive impairment.

*p < 0.05.

**FIGURE 4:**
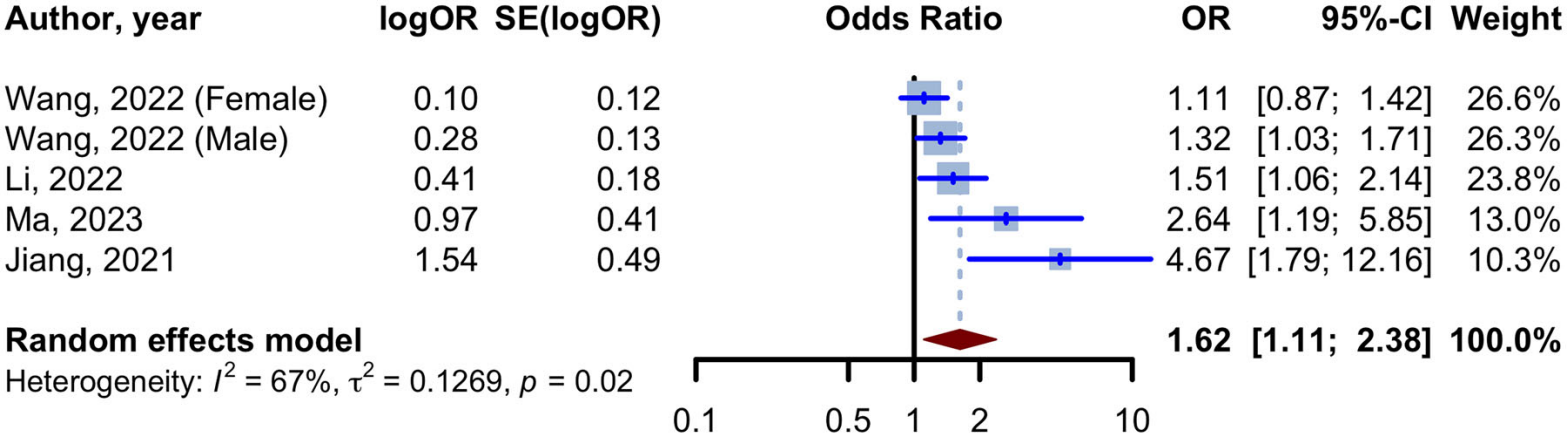
Forest plot for meta‐analysis of adjusted odds ratio for the relationship between TyG index (Quartile 4 vs. Quartile 1) and cognitive decline.

We next examined six studies comparing the quartiles (Q) of the TyG index (Hong, Han, and Park 2021; Jiang et al. 2021; S. Li, Deng, and Zhang 2022; Ma et al. 2023; Sun et al. 2023; Wang et al. 2022). Of these, the study from Hong et al. (Hong, Han, and Park 2021) and Sun et al. (Sun et al. 2023) reported HRs for comparing Q4 with Q1 and were not added to the meta‐analysis of ORs. In their population‐based study, Hong and colleagues (Hong, Han, and Park 2021) found a higher incidence of all‐cause dementia in Q2, Q3, and Q4 of the TyG index compared to Q1 (Q2: aHR 1.04 [95% CI 1.02 to 1.05], Q3: aHR1.07 [95% CI 1.05 to 1.09], Q4: aHR 1.14 [95% CI 1.12 to 1.16]). For their part, Sun and colleagues evaluated AD incidence in a population‐based study (Sun et al. 2023). Interestingly, these authors observed that only patients in the Q3 of the TyG index had a significantly higher incidence of AD than those in the Q1 (aHR 1.69, 95% CI 1.02 to 2.81). Next, we compared Q of the TyG index in fully adjusted models described in four of the six studies reporting ORs (Jiang et al. 2021; S.Li, Deng, and Zhang 2022; Ma et al. 2023; Wang et al. 2022). As shown in the forest plot in Figure 4, our analysis revealed that the fourth and first quartiles of the TyG index showed significant odds of cognitive decline (aOR 1.62, 95% CI 1.11 to 2.38, *p* = 0.013, I2: 67%). On the other hand, a similar significant OR was obtained by pooling unadjusted ORs (OR 3.63, 95% CI 1.12 to 11.74, *p* = 0.032, *I* ^2^: 92%), as illustrated in Figure S4. Finally, the adjusted OR/HR for comparison of cognitive decline between groups of TyG index is available in Table 3.

### 3.4 Diagnostic Ability of TyG for Cognitive Decline

**FIGURE 5:**
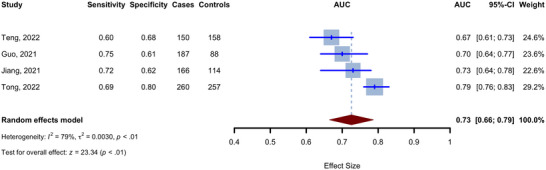
Forest plot for pooled AUC for diagnosis of cognitive decline.

**FIGURE 6:**
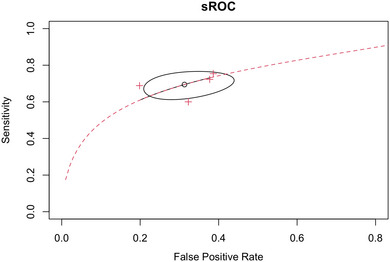
Summary receiver operative curve for measurement of TyG for assessment of cognitive decline.

The diagnostic ability of the TyG index for cognitive decline was assessed in four studies (Guo et al. 2021; Jiang et al. 2021; Teng et al. 2022; Tong et al. 2022). Pooled AUC by random effect meta‐analysis is shown as a forest plot in Figure 5 (AUC 0.73, 95% CI 0.66 to 0.79, *p* < 0.001). The heterogeneity was high in this analysis (*I* ^2^: 79%). Obtaining AUC by pooling sensitivities and specificities observed in each study resulted in an overall AUC of 0.74. Also, a sensitivity of 0.695 (95% CI 0.629 to 0.753) and a specificity of 0.687 (95% CI 0.587 to 0.772) were observed, as shown in Figure 6 and Figure S5.

## 4 Discussion

In this study, we performed a systematic review and meta‐analysis to establish a correlation between cognitive decline and the TyG index. We examined 17 studies identified after literature screening and included them because they respected our inclusion criteria described in Section 2. The main results obtained following our analysis are that (1) individuals experiencing cognitive decline exhibited more elevated levels of the TyG index than those with normal cognitive function, (2) an escalation in the TyG index corresponds to a substantially heightened risk of cognitive decline, and (3) higher TyG indexes are associated with cognitive decline and dementia, even in nondiabetic patients, supporting the central independent pathogenic role of IR.

IR and impaired insulin secretion are often present in patients with type 2 diabetes and those with poor glucose tolerance (Zhao et al. 2023). However, aside from diabetes, IR spawns, or coexists with, other conditions like cardiovascular disease, cancer, nonalcoholic fatty liver disease (NAFLD), and other metabolic diseases (Zhao et al. 2023). In addition to these, IR appears to be directly associated with the risk of cognitive decline and dementia (Kim and Arvanitakis 2023). Therefore, understanding IR clearly and exploring innovative therapeutic and diagnostic approaches to reduce the risk of IR‐related disease burden is utterly needed. In this regard, the reduction of glycemic and lipid profiles of patients at cognitive risk using novel anti‐diabetic agents such as sodium‐glucose cotransporter inhibitors (SGLT2i), which could lead to a decrease in TyG index as a marker of IR, leads to better management of dementia (Lardaro et al. 2024; Mui et al. 2021).

IR can be measured using its gold standard, namely the hyperinsulinemic‐positive glucose clamp test (HEGC). However, its clinical application remains poor due to its limitations and complexity. Hence, other less invasive tools have been implemented, such as the homeostatic model assessment (HOMA‐IR) and the TyG index (X. Li et al. 2023). Compared to the HOMA‐IR, the TyG index has gained popularity as it is a more cost‐effective alternative and easy‐to‐measure surrogate marker of the IR (Kang et al. 2017; Minh et al. 2021; Wallace, Levy, and Matthews 2004). Moreover, the clinical usage of the TyG index has been supported by several studies (including meta‐analysis) showing the potential diagnostic and prognostic role of this marker in various IR‐related diseases (Sánchez‐Íñigo et al. 2016; Khalaji et al. 2023; Okamura et al. 2020; Hong, Han, and Park 2021; Behnoush et al. 2024; Azarboo et al. 2024). Clinicians could benefit from measuring this easily calculated index in order to assess the risk of cognitive decline in high‐risk populations. Also, due to the ease of TyG index measurement, assessing TyG level could be useful for screening the general population for not only preventing metabolic conditions but also other nonmetabolic diseases such as cognitive decline.

Importantly, as discussed by the 2020 Lancet Commission on dementia prevention, intervention, and care, there are at least 12 modifiable risk factors that might prevent or delay up to 40% of cases of dementia, and for most of these, like hypertension, depression, smoking, diabetes, sleep, diet, obesity, and alcohol, an association has been described with IR or the TyG index (Hong, Han, and Park 2021; Gao et al. 2023; Korkmaz et al. 2023; Pei et al. 2023; Kim et al. 2023; Chen, Gu, and Huang 2023; Baek et al. 2021). Since the TyG index is a novel index and there are still a limited number of studies on the association of TyG and cognitive decline, as shown in our findings, there is a need for future studies on this to better elucidate this connection.

Our findings support the notion that higher TyG index levels are associated with a risk of decline, aligning with most previous studies. Nonetheless, some studies have not found a connection between insulin resistance and cognitive function. For instance, one study utilizing HOMA2 IR to calculate the insulin resistance index found no association between HOMA2 IR and cognitive function in individuals with type 2 diabetes (Xia et al. 2020; Geijselaers et al. 2017). Since HOMA2‐IR is primarily intended to assess the effects of peripheral insulin resistance on organs such as the liver and skeletal muscle, some researchers argue that it may not accurately indicate brain insulin resistance (Banks, Owen, and Erickson 2012). Thus, more research is necessary to determine whether the TyG index and brain insulin resistance are related.

Of note, our study aligns with most previous studies and confirms that higher TyG index levels are associated with a risk of cognitive decline. Importantly, such an association appears to be independent of the presence of diabetes. Indeed, of the seventeen studies examined, just two (Teng et al. 2022; Tong et al. 2022) exclusively analyzed cognitive decline and TyG index in type 2 diabetic patients, with the remaining studies assessing this association in the general population or patients with dementia/cognitive impairment or cerebral small vessel disease (Table S3). In line with our analysis, the study by Hong et al. (Hong, Han, and Park 2021) in a large population of over 5 million participants enrolled during a median follow‐up of 7.21 years demonstrated that the TyG index was associated with an increased risk of dementia that was independent of traditional risk factors.

Despite this, the correlation between dementia and IR was not demonstrated in all studies, especially in those evaluating IR with the HOMA‐IR tool. Importantly, heterogeneities in populations, study designs, and variability in methods of cognitive assessment and IR may explain these inconsistencies. However, additional research is imperative, especially in subgroups, to better understand the relationship between IR and cognitive decline and then validate and refine the TyG index's diagnostic ability.

## 5 Conclusion

Our study supports a significant association between cognitive decline and high TyG index values. We revealed the heightened risk of cognitive decline with an increased TyG index and underscored the potential diagnostic capability of this surrogate marker of IR. The assessment of the TyG index's predictive capacity for cognitive decline yielded promising outcomes and highlighted its diagnostic potential with an impressive overall AUC of 0.74, a sensitivity of 0.695, and a specificity of 0.68. Moreover, our multivariate meta‐regression analysis revealed a significant association between the observed pooled estimate and the publication year, sample size, and male ratio. Of note, several questions remain open. Among these, one big question is how IR is mechanistically related to cognitive decline. Therefore, more research in the field, especially preclinical studies, may be helpful in better understanding how IR is associated with cognitive decline and dementia. These studies will help clinicians tailor specific interventions and diagnostic approaches to reduce the burden of dementia.

## Author Contributions

**Elina Ghondaghsaz**: conceptualization, formal analysis, writing–original draft, visualization. **Amirmohammad Khalaji**: conceptualization, writing–original draft, visualization, formal analysis. **Mehrdad Mahalleh**: writing–original draft, writing–review and editing. **Mahdi Masrour**: writing–original draft, writing–review and editing. **Parsa Mohammadi**: writing–original draft, writing–review and editing. **Alessandro Cannavo**: writing–original draft, data curation. **Amir Hossein Behnoush**: writing–original draft, writing–review and editing, supervision.

## Ethics Statement

The authors have nothing to report.

## Consent

The authors declare no conflicts of interest.

## Conflict of Interest

The authors declare no conflicts of interest.

### Peer Review

The peer review history for this article is available at https://publons.com/publon/10.1002/brb3.70131.

## Supporting information



Supporting Information

## Data Availability

Data sharing is not applicable to this article as no new data were created or analyzed in this study.
