# Supplementary material for: Exploring the Association Between Cognitive Decline and Triglyceride‐Glucose Index: A Systematic Review and Meta‐Analysis
Source: Brain Behav. 2024 Oct 31;14(11):e70131. doi: 10.1002/brb3.70131 (PMC11527841; doi:10.1002/brb3.70131)
Supplement: Supplementary file 1 — Supporting Information [file BRB3-14-e70131-s001.docx]

**Supplementary Materials**

***Supplementary Table 1.*** *The search queries used for each database and the search results*

| Query | | Results (No.)  25 October 2023 |
| --- | --- | --- |
| **PubMed** | | |
| #1 | (“TyG”[tiab] OR “triglyceride glucose”[tiab] OR “triglyceride-glucose”[tiab] OR “triglyceride-glucose index”[tiab]) | 1,447 |
| #2 | (“dementia”[tiab] OR “dement*”[tiab] OR “cogniti*”[tiab] OR “neurocogn*”[tiab] OR “VaD”[tiab] OR “Alzheimer”[tiab] OR “Alzheimer’s”[tiab] OR “AD”[tiab] OR “FTD”[tiab] OR “frontotemporal lobar degeneration”[tiab] OR “FTLD”[tiab] OR “frontotemporal degenerati*”[tiab] OR “mild cognitive impairment”[tiab] OR “MCI”[tiab] OR “memory loss”[tiab] OR “amnestic”[tiab] OR “amnesia”[tiab] OR “Lewy bod*”[tiab] OR “creutzfeldt-jakob”[tiab] OR ʺDementiaʺ[mh] OR ʺCognitive Dysfunctionʺ[mh]) | 901,430 |
| #3 | #1 AND #2 | 31 |
| **Embase** | | |
| #1 | (“TyG”:ti,ab,kw OR “triglyceride glucose”:ti,ab,kw OR “triglyceride-glucose”:ti,ab,kw OR “triglyceride-glucose index”:ti,ab,kw) | 1,860 |
| #2 | (“dementia”:ti,ab,kw OR “dement*”:ti,ab,kw OR “cogniti*”:ti,ab,kw OR “neurocogn*”:ti,ab,kw OR “VaD”:ti,ab,kw OR “Alzheimer”:ti,ab,kw OR “AD”:ti,ab,kw OR “FTD”:ti,ab,kw OR “frontotemporal lobar degeneration”:ti,ab,kw OR “FTLD”:ti,ab,kw OR “frontotemporal degenerati*”:ti,ab,kw OR “mild cognitive impairment”:ti,ab,kw OR “MCI”:ti,ab,kw OR “memory loss”:ti,ab,kw OR “amnestic”:ti,ab,kw OR “amnesia”:ti,ab,kw OR “Lewy bod*”:ti,ab,kw OR “creutzfeldt-jakob”:ti,ab,kw) | 1,197,506 |
| #3 | #1 AND #2 | 38 |
| **Web of Science** | | |
| #1 | (TS=“TyG” OR TS=“triglyceride glucose” OR TS=“triglyceride-glucose” OR TS=“triglyceride-glucose index”) | 1,417 |
| #2 | (TS=“dementia” OR TS=“dement*” OR TS=“cogniti*” OR TS=“neurocogn*” OR TS=“VaD” OR TS=“Alzheimer” OR TS=“AD” OR TS=“FTD” OR TS=“frontotemporal lobar degeneration” OR TS=“FTLD” OR TS=“frontotemporal degenerati*” OR TS=“mild cognitive impairment” OR TS=“MCI” OR TS=“memory loss” OR TS=“amnestic” OR TS=“amnesia” OR TS=“Lewy bod*” OR TS=“creutzfeldt-jakob”) | 1,206,163 |
| #3 | #1 AND #2 | 32 |
| **SCOPUS** | | |
| #1 | (TITLE-ABS-KEY(“TyG”) OR TITLE-ABS-KEY(“triglyceride glucose”) OR TITLE-ABS-KEY(“triglyceride-glucose”) OR TITLE-ABS-KEY(“triglyceride-glucose index”)) | 2,510 |
| #2 | (TITLE-ABS-KEY(“dementia”) OR TITLE-ABS-KEY(“dement*”) OR TITLE-ABS-KEY(“cogniti*”) OR TITLE-ABS-KEY(“neurocogn*”) OR TITLE-ABS-KEY(“VaD”) OR TITLE-ABS-KEY(“Alzheimer”) OR TITLE-ABS-KEY(“AD”) OR TITLE-ABS-KEY(“FTD”) OR TITLE-ABS-KEY(“frontotemporal lobar degeneration”) OR TITLE-ABS-KEY(“FTLD”) OR TITLE-ABS-KEY(“frontotemporal degenerati*”) OR TITLE-ABS-KEY(“mild cognitive impairment”) OR TITLE-ABS-KEY(“MCI”) OR TITLE-ABS-KEY(“memory loss”) OR TITLE-ABS-KEY(“amnestic”) OR TITLE-ABS-KEY(“amnesia”) OR TITLE-ABS-KEY(“Lewy bod*”) OR TITLE-ABS-KEY(“creutzfeldt-jakob”)) | 1,853,253 |
| #3 | #1 AND #2 | 65 |
| ***TOTAL RECORDS*** | | ***166*** |
| ***TOTAL RECORDS AFTER REMOVING DUPLICATES*** | | ***100*** |

***Supplementary Table 2.*** *Qualities of included studies based on NOS*

| **Study** | **Selection** | | | | **Comparability** | **Outcome** | | | **Overall**  **Score** |
| --- | --- | --- | --- | --- | --- | --- | --- | --- | --- |
|  | **Representation of exposed cohort** | **Selection of the non-exposed cohort** | **Ascertainment of exposure** | **Outcome of interest presence** |  | **Assessment of outcome** | **Sufficient length of follow-up** | **Loss to follow-up** |  |
| Faqih et al. (2021) (1) | * | * | * | * | - | * | - | * | 6 |
| Gentreau et al. (2022) (2) | * | * | * | * | - | * | *  (7 years) | * | 7 |
| Guo et al. (2021) (3) | * | * | * | * | - | * | - | * | 6 |
| Hong et al. (2021) (4) | * | * | * | * | - | * | *  (Mean 7.2 years) | * | 7 |
| Huang et al. (2022) (5) | * | * | * | * | - | * | - | * | 6 |
| Jiang et al. (2021) (6) | * | * | * | * | - | * | - | * | 6 |
| Li et al. (2022) (7) | * | * | * | * | - | * | *  (4 years) | * | 7 |
| Liu et al. (2023) (8) | * | * | * | * | - | * | - | * | 6 |
| Ma et al. (2023) (9) | * | * | * | * | - | * | - | * | 6 |
| Seo et al. (2023) (10) | * | * | * | * | ** | * | - | * | 8 |
| Sun et al. (2023) (11) | * | * | * | * | - | * | *  (Median 13.8 years) | * | 7 |
| Teng et al. (2022) (12) | * | * | * | * | - | * | - | * | 6 |
| Tian et al. (2023) (13) | * | * | * | * | - | * | - | * | 6 |
| Tian et al. (2023) (14) | * | * | * | * | - | * | - | * | 6 |
| Tong et al. (2022) (15) | * | * | * | * | - | * | - | * | 6 |
| Wang et al. (2022) (16) | * | * | * | * | - | * | *  (Every 2-3 years) | * | 7 |
| Weyman-Vala et al. (2022) (17) | * | * | * | * | ** | * | - | * | 8 |

***Supplementary Table 3.*** *Adjusted covariates in multivariable models*

| **Study (year)** | **Population** | **Outcome** | **OR/HR** | **Covariates** |
| --- | --- | --- | --- | --- |
| Faqih et al. (2021) (1) | Patients with AD or memory loss | AD | OR | Age, gender, body mass index, co-morbidities, insulin intake, and HbA1c |
| Guo et al. (2021) (3) | Elderly patients with CSVD with or without VCI | VCI | OR | Age, level of education, low-density lipoprotein cholesterol, homocysteine, uric acid, and total CSVD burden |
| Hong et al. (2021) (4) | Population-based study | All-cause dementia | HR | Age, sex, smoking status, alcohol consumption, physical activity, low income, body mass index, hypertension, and total cholesterol level |
| Jiang et al. (2021) (6) | CSVD patients with or without VCI | VCI | OR | Age, sex, body mass index, diabetes, hypertension, education level, high-density lipoprotein cholesterol, low-density lipoprotein cholesterol, high sensitivity C-reactive protein, HbA1c, interleukin-34, modified Rankin Scale, and Barthel Index |
| Li et al. (2022) (7) | Population-based study | Cognitive decline | OR | Age, sex, educational levels, smoking status, alcohol consumption, physical activity, body mass index, history of hypertension, and total cholesterol |
| Ma et al. (2023) (9) | Population-based study | Cognitive impairment | OR | Age, sex, smoking, drinking, physical exercise habits, cardiovascular disease, hypertension, diabetes mellitus, taking hypoglycemic drugs and lipid-lowering drugs, total cholesterol, body mass index, and waist circumference |
| Sun et al. (2023) (11) | Population-based study | AD | HR | Age at Exam 7, sex, education level (lower or higher than high school), body mass index, current smoking, physical activity index, systolic blood pressure (categorical variable, >140mmHg or ≤140mmHg), cardiovascular disease, antihypertensives, hypoglycemic therapy, and lipid-lowering therapy |
| Teng et al. (2022) (12) | Elderly patients with T2DM | Cognitive impairment | OR | Age, education, systolic blood pressure, HbA1c, history of stroke, high-density lipoprotein cholesterol, serum total homocysteine, severe CSVD burden, and insulin or metformin usage |
| Tian et al. (2023) (13) | Population-based study | All-cause dementia | OR | Age, sex, education, APOE genotype, body mass index, current smoking, alcohol consumption, hypertension, hypercholesterolemia, and if applicable, for cardiovascular diseases (coronary heart disease, heart failure, atrial fibrillation, and stroke) and use of glucose-lowering drugs or insulin injection |
| Tong et al. (2022) (15) | T2DM patients with or without MCI | MCI | OR | Age, gender, smoking, drinking history, duration of diabetes, education level, total cholesterol, HbA1c, diabetic nephropathy, fatty liver, insulin use, and statins use |
| Wang et al. (2022) (16) | Population-based study | Cognitive decline | OR | Age, sex, education, marriage, residence, leisure time social activity, health insurance status, alcohol use, smoking status, hypertension, diabetes, and body mass index |

OR: odds ratio, HR: hazard ratio, CSVD: cerebral small vessel disease, VCI: vascular cognitive impairment, AD: Alzheimer’s disease, MCI: mild cognitive impairment, T2DM: type 2 diabetes mellitus

**
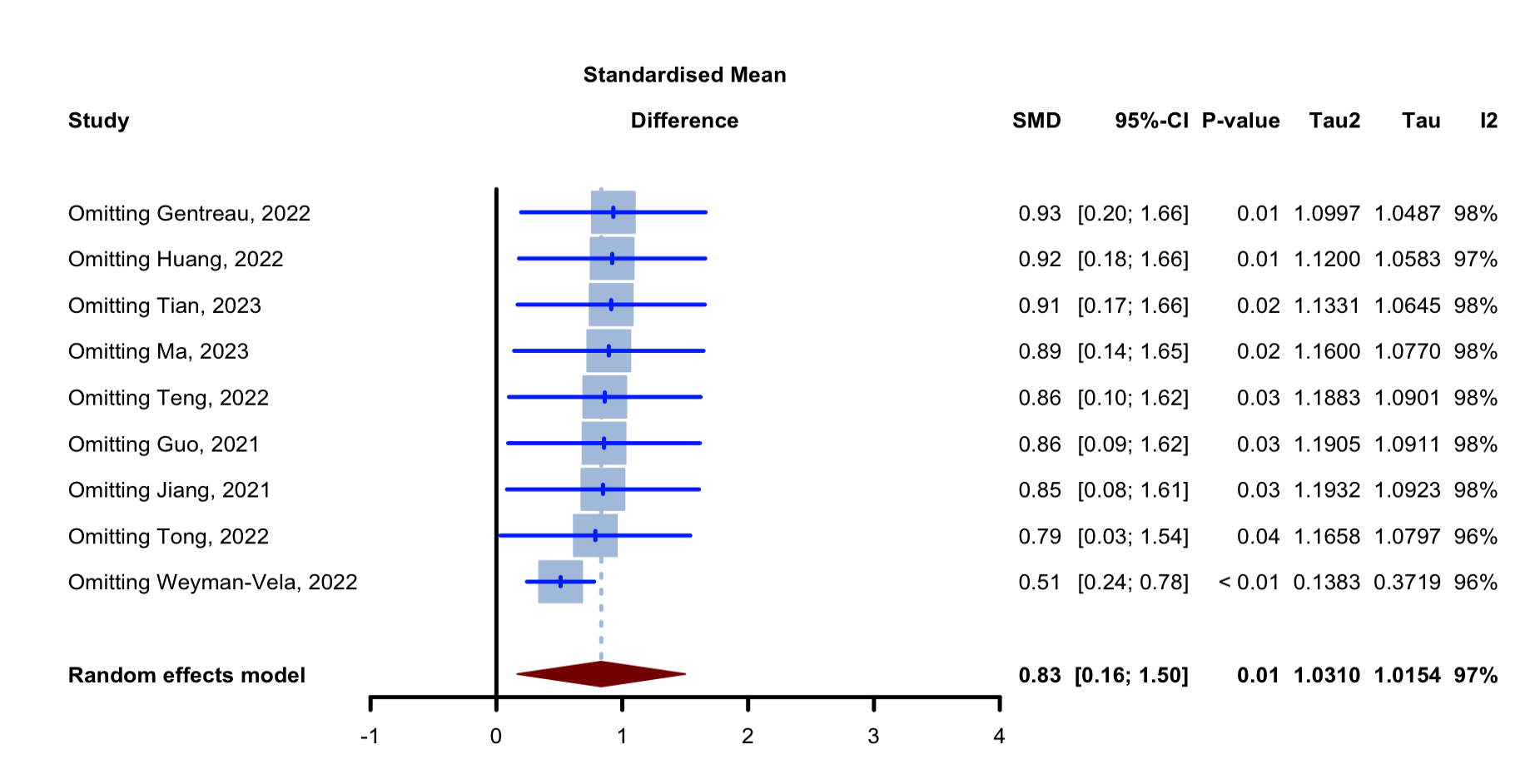
**

***Supplementary Figure 1.*** *Sensitivity analysis by leave-one-out method for meta-analysis of TyG levels in patients with cognitive decline vs. controls*


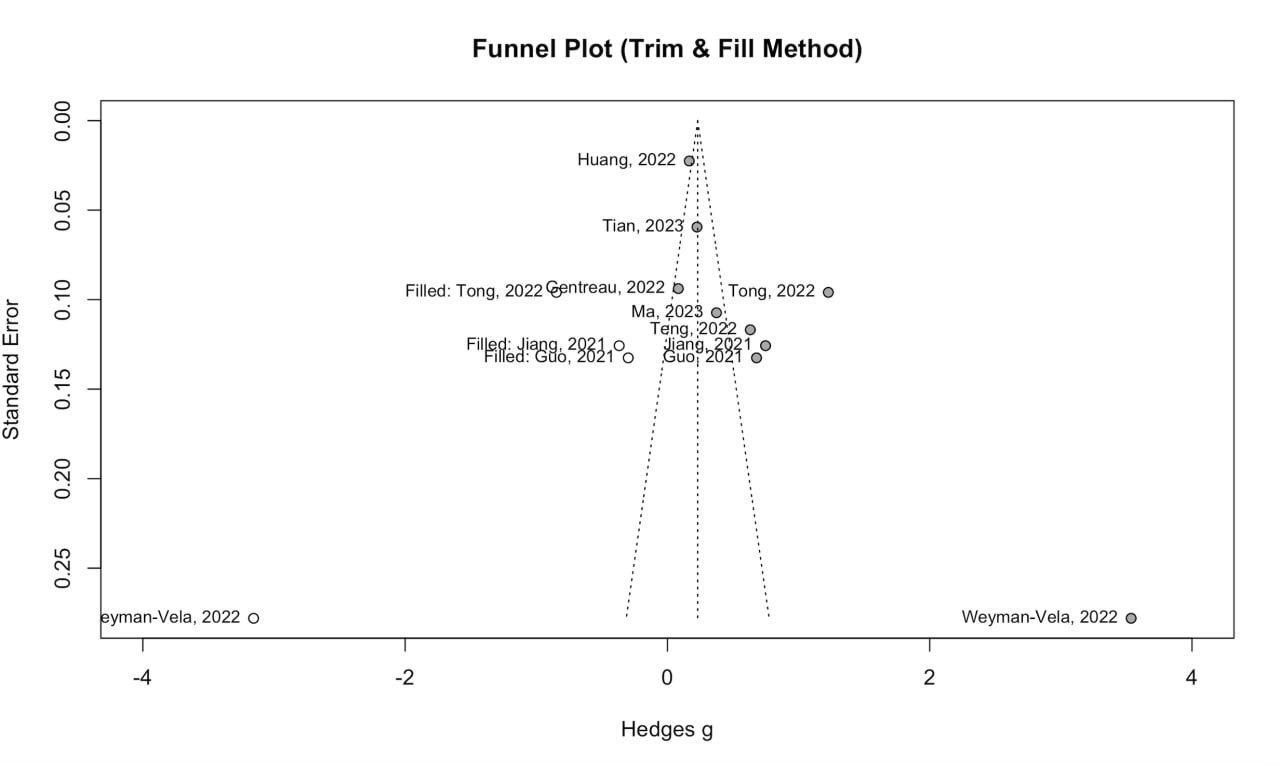


***Supplementary Figure 2.*** *Funnel plot for meta-analysis of TyG levels in patients with cognitive decline vs. controls*

**
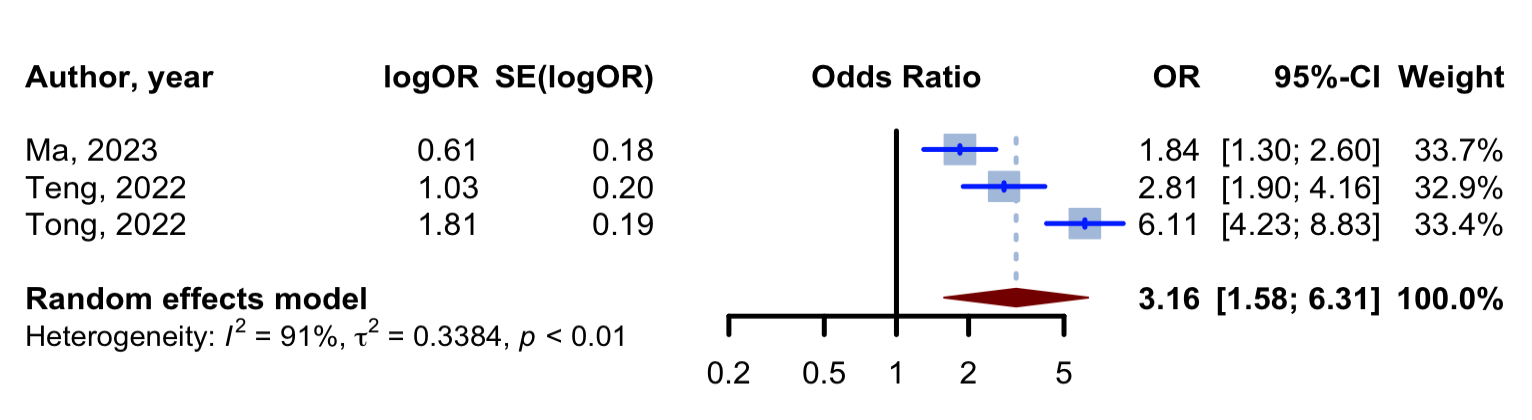
**

***Supplementary Figure 3.*** *Forest plot for meta-analysis of unadjusted odds ratio for the relationship between TyG index (1-unit increase) and cognitive decline*

**
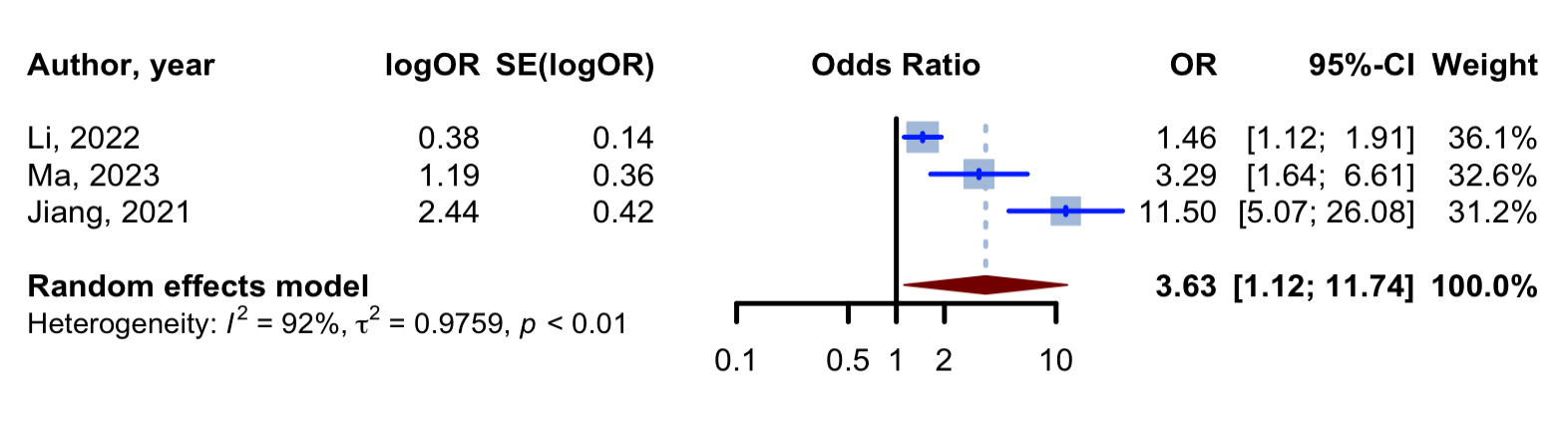
**

***Supplementary Figure 4.*** *Forest plot for meta-analysis of unadjusted odds ratio for the relationship between TyG index (Quartile 4 vs. Quartile 1) and cognitive decline*


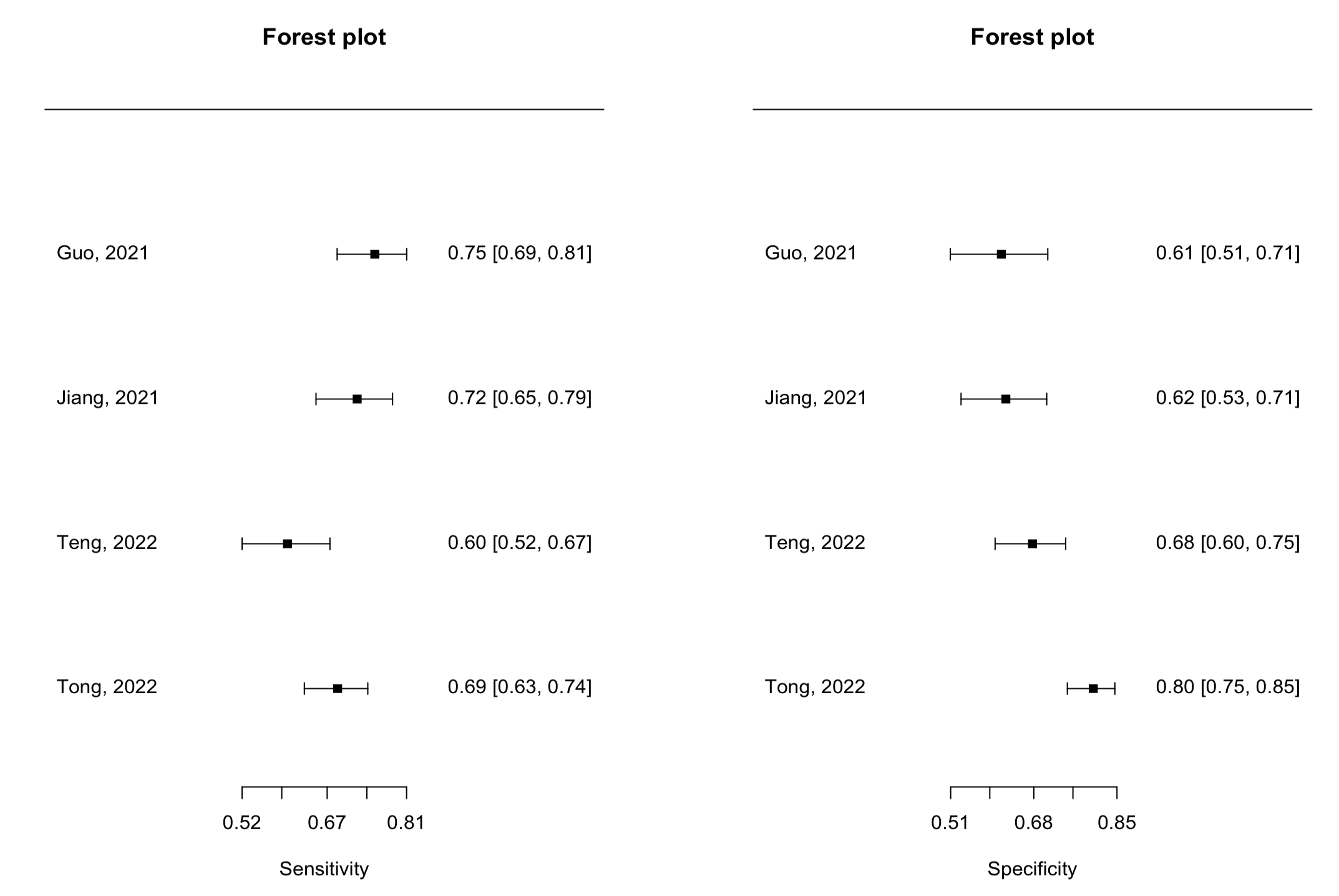


***Supplementary Figure 5.*** *Pooled sensitivity and specificity of TyG index for diagnosis of cognitive decline*

**References:**

1. Faqih NT, Ashoor AF, Alshaikh SA, Maglan AF, Jastaniah N. Association of Alzheimer's Disease and Insulin Resistance in King Abdulaziz Medical City, Jeddah. Cureus. 2021;13(11):e19811.

2. Gentreau M, Reynes C, Sabatier R, Maller JJ, Meslin C, Deverdun J, et al. Glucometabolic Changes Are Associated with Structural Gray Matter Alterations in Prodromal Dementia. J Alzheimers Dis. 2022;89(4):1293-302.

3. Guo X, Lu Z, Cao Z, Li X, Zhu X. Correlation between triglyceride-glucose index and vascular cognitive impairment in elderly patients with cerebral small vessel disease. Chi J Gen Prac. 2021;20(9):984-9.

4. Hong S, Han K, Park CY. The insulin resistance by triglyceride glucose index and risk for dementia: population-based study. Alzheimers Res Ther. 2021;13(1):9.

5. Huang SH, Chen SC, Geng JH, Wu DW, Li CH. Metabolic Syndrome and High-Obesity-Related Indices Are Associated with Poor Cognitive Function in a Large Taiwanese Population Study Older than 60 Years. Nutrients. 2022;14(8).

6. Jiang T, Zhou Y, Zhang D, Cao Z, Bian L, Ni Y, et al. Association of Serum Interleukin-34 and Insulin Resistance with Cognitive Impairment in Patients with Cerebral Small Vessel Disease. Curr Neurovasc Res. 2021;18(4):446-55.

7. Li SQ, Deng X, Zhang YM. The Triglyceride-Glucose Index Is Associated with Longitudinal Cognitive Decline in a Middle-Aged to Elderly Population: A Cohort Study. JOURNAL OF CLINICAL MEDICINE. 2022;11(23).

8. Liu X, Hong Y, Huai C, Feng L, Li W, Li R, et al. Correlation of related indexes of blood lipid and insulin resistance with cognition scores in middle-aged and elderly people with mild cognitive impairment. Chin J Health Manag. 2023;17(1):13-8.

9. Ma YM, Wei S, Dang LJ, Gao L, Shang SH, Hu NW, et al. Association between the triglyceride-glucose index and cognitive impairment in China: a community population-based cross-sectional study. NUTRITIONAL NEUROSCIENCE. 2023.

10. Seo MW, Gann J, Lee JM, Heffernan KS, Kim JY, Jung HC. Potential impact of metabolic syndrome on cognitive function in US firefighters. Front Public Health. 2023;11:1150121.

11. Sun J, Xie Z, Wu Y, Liu X, Ma J, Dong Y, et al. Association of the Triglyceride-Glucose Index With Risk of Alzheimer's Disease: A Prospective Cohort Study. Am J Prev Med. 2023.

12. Teng Z, Feng J, Dong Y, Xu J, Jiang X, Chen H, et al. Triglyceride glucose index is associated with cerebral small vessel disease burden and cognitive impairment in elderly patients with type 2 diabetes mellitus. Front Endocrinol (Lausanne). 2022;13:970122.

13. Tian N, Fa W, Dong Y, Liu R, Liu C, Liu K, et al. Triglyceride-glucose index, Alzheimer's disease plasma biomarkers, and dementia in older adults: The MIND-China study. Alzheimers Dement (Amst). 2023;15(2):e12426.

14. Tian N, Song L, Hou T, Fa W, Dong Y, Liu R, et al. Association of Triglyceride-Glucose Index With Cognitive Function and Brain Atrophy: A Population-Based Study. Am J Geriatr Psychiatry. 2023.

15. Tong XW, Zhang YT, Yu ZW, Pu SD, Li X, Xu YX, et al. Triglyceride Glucose Index is Related with the Risk of Mild Cognitive Impairment in Type 2 Diabetes. Diabetes Metab Syndr Obes. 2022;15:3577-87.

16. Wang K, Xu L, Liu LL, Zhan SY, Wang SF, Song YF. Sex differences in the association between the change in triglyceride-glucose index and cognitive decline: A population-based cohort study. JOURNAL OF AFFECTIVE DISORDERS. 2022;316:42-9.

17. Weyman-Vela Y, Simental-Mendía LE, Camacho-Luis A, Gamboa-Gómez CI, Guerrero-Romero F. The Triglycerides and Glucose Index Is Associated with Mild Cognitive Impairment in Older Adults. Endocr Res. 2022;47(2):89-93.
